# Development and Application of a Metaverse-Based Social Skills Training Program for Children With Autism Spectrum Disorder to Improve Social Interaction: Protocol for a Randomized Controlled Trial

**DOI:** 10.2196/35960

**Published:** 2022-06-08

**Authors:** JooHyun Lee, Tae Seon Lee, SeungWoo Lee, JiHye Jang, SuYoung Yoo, YeJin Choi, Yu Rang Park

**Affiliations:** 1 Department of Biomedical Systems Informatics Yonsei University College of Medicine Seoul Republic of Korea; 2 Department of Neurosurgery, Severance Hospital Yonsei University College of Medicine Seoul Republic of Korea; 3 Graduate School of Information and Communication Technology Ajou University Suwon Republic of Korea; 4 DoBrain Co, Ltd Seoul Republic of Korea

**Keywords:** metaverse, social skills, Autism, ASD, digital therapy, Roblox, RCT, social skill, social interaction, human interaction, child, youth, development, wearable, biometric, communication, digital technology, eHealth, mhealth, stress, emotional change, online platform

## Abstract

**Background:**

Autism spectrum disorder (ASD) is characterized by abnormalities in social communication and limited and repetitive behavioral patterns. Children with ASD who lack social communication skills will eventually not interact with others and will lack peer relationships when compared to ordinary people. Thus, it is necessary to develop a program to improve social communication abilities using digital technology in people with ASD.

**Objective:**

We intend to develop and apply a metaverse-based child social skills training program aimed at improving the social interaction abilities of children with ASD aged 7-12 years. We plan to compare and analyze the biometric information collected through wearable devices when applying the metaverse-based social skills training program to evaluate emotional changes in children with ASD in stressful situations.

**Methods:**

This parallel randomized controlled study will be conducted on children aged 7-12 years diagnosed with ASD. A metaverse-based social skills training program using digital technology will be administered to children who voluntarily wish to participate in the research with consent from their legal guardians. The treatment group will participate in the metaverse-based social skills training program developed by this research team once a week for 60 minutes per session for 4 weeks. The control group will not intervene during the experiment. The treatment group will use wearable devices during the experiment to collect real-time biometric information.

**Results:**

The study is expected to recruit and enroll participants in March 2022. After registering the participants, the study will be conducted from March 2022 to May 2022. This research will be jointly conducted by Yonsei University and Dobrain Co Ltd. Children participating in the program will use the internet-based platform.

**Conclusions:**

The metaverse-based Program for the Education and Enrichment of Relational Skills (PEERS) will be effective in improving the social skills of children with ASD, similar to the offline PEERS program. The metaverse-based PEERS program offers excellent accessibility and is inexpensive because it can be administered at home; thus, it is expected to be effective in many children with ASD. If a method can be applied to detect children's emotional changes early using biometric information collected through wearable devices, then emotional changes such as anxiety and anger can be alleviated in advance, thus reducing issues in children with ASD.

**Trial Registration:**

Clinical Research Information Service KCT0006859; https://tinyurl.com/4r3k7cmj

**International Registered Report Identifier (IRRID):**

PRR1-10.2196/35960

## Introduction

Autism spectrum disorder (ASD) is a complex developmental condition that involves persistent challenges in social interaction, speech, and nonverbal communication, with restricted or repetitive behaviors [1]. In You’s study, deficits in social-emotional exchange, such as social communication disorder in interactions covering the present or past developmental period, are discussed along with deficits in nonverbal communication in social interaction and relationship formation appropriate to the developmental level. Impaired social functioning, which often requires lifetime treatment, is a characteristic of ASD [2]. Typically, school-aged children and adolescents acquire and develop the basic rules of social etiquette through observation of peer behavior or specific parental instruction [3]; however, some school-aged children with developmental disabilities, or some children and adolescents with ASD, have difficulties in acquiring the social etiquette necessary for peer relationships and they require additional intervention. As they lack social interaction skills, they are more likely to be isolated from their peers, making it evident that the etiquette necessary for relationships is lacking [4]. Furthermore, without treatment, many adults with ASD will have fewer interactions and friendships than an average person [5]. Imparting people with ASD the skills to make and retain friends to people with ASD is expected to have a lasting and significant impact on their lives. The prevalence of ASD has increased worldwide. Hong and Lee investigated the economic burden of ASD in South Korea using a nationally representative data source. The direct medical, nonmedical, and indirect costs resulting from ASD were estimated. The total prevalence was 5.04 (per 100,000 people) in 2008 and 10.97 in 2015. The economic cost of ASD was estimated at US $2,700,596 in 2008 and US $9,645,503 in 2015. The results of this study suggest that the increase in economic costs is greater than the increase in the prevalence [6]. Consultation with child psychiatrists for ASD diagnosis, psychological evaluation, and treatment, including ASD testing, are primarily available in cities, necessitating long waiting times and incurring high costs. Furthermore, even after diagnosis, treatments including speech therapy, psychotherapy, play therapy, and occupational therapy are centrally operated in cities, and treatment costs are high [7]. Therefore, appropriate treatment for many children with ASD is delayed. Furthermore, it is difficult for children from low-income families to receive timely diagnosis and treatment because of the high cost of treatment in facilities concentrated in cities.

Various treatment strategies for ASD include educational and behavioral interventions that target its core features. Well-designed interventions, such as risperidone and aripiprazole, have been reported to have strong and beneficial effects [8,9]. The Program for the Education and Enrichment of Relational Skills (PEERS) is a manualized evidence-based social skills program developed from ASD intervention programs [10]. The PEERS program used in this study has been designed to improve social and friendship skills in adolescents with high-function ASD. The results of a previous randomized controlled study examining the efficacy of PEERS in improving social abilities and friendship skills in high-functioning adolescents with ASD showed that the group involved in the program significantly improved its knowledge of social skills compared to the control group that was not part of the program. The frequency of meeting with friends increased and the overall social skills improved [11,12]. The Korean version of the PEERS social skills program appears to be effective for adolescents with ASD in Korea after modest adjustments for cultural differences. In a randomized controlled trial (RCT), participants who received the PEERS treatment showed significant improvement in social skill knowledge, interpersonal skills, state anxiety or depressive symptoms, as well as a decrease in ASD symptoms [2]. For adolescents with ASD who had previously received the PEERS program face to face, the program was suspended or postponed because of the COVID-19 outbreak, owing to the restrictions declared by many government statutes [13]. Children with ASD require continuous long-term training to improve their cognitive development and behavior. Due to the lack of social reciprocity and communication, special education teachers, regular training activities, and training locations are relatively fixed. However, the sudden COVID-19 outbreak disrupted the familiar and routine training activities of preschoolers with ASD, and limitations in the children’s physical environment may exacerbate behavioral problems [14]. Therefore, there is an increasing need to shift traditional therapeutic environments from face-to-face learning to internet-based play. According to the World Health Organization, telemedicine refers to the use of communication and virtual technologies to provide health care [15]. The advantages of telemedicine include the ability to receive treatment in a comfortable environment, ensuring continuity of care, low cost, high accessibility, and easy dissemination. Recent studies have shown that telemedicine interventions can improve the behavior of children with ASD [14,16-18]. Education was delivered using virtual reality (VR) technology to increase sociality for children and adults with ASD, and as a result, previous studies have reported improved sociality [19-21]. We designed the study to use a metaverse-based interactive game platform rather than VR equipment. Roblox, MineCraft, Whyville, and Zepeto are internet-based virtual world game platforms on which users can socialize, be creative, and play using their imagination [22,23]. Internet-based virtual world game platforms provide various types of cooperative activities in which children and adolescents can participate. Collaborative activities include solving problems and challenges, forming teams to execute missions through collaboration and organization, creating and decorating avatars, and practicing digital literacy skills, such as coding and writing. Internet-based multiplayer games can strengthen healthy communication and social connections as well as alleviate social isolation [24]. Playing games on internet-based gaming platforms can help foster a sense of belonging and develop friendships that are essential for the social and emotional development of children and adolescents. Developing a social skills training program using an internet-based virtual world game will be effective in improving social skills and will be an important treatment method during the COVID-19 pandemic situation, especially in the case of children and adolescents with ASD.

To improve the social skills of children with ASD, it is necessary to predict their anxiety early by collecting biometric information using wearable devices. Anxiety is a common problem in children, adolescents, and adults with ASD. Anxiety caused by emotion regulation impairments in children with ASD can lead to many behavioral problems, such as aggression and irritability [25]. Furthermore, problematic behavior, including self-harm in children with ASD, can be a significant barrier to accessing community services, including education, and can affect social improvement by limiting peer group formation or social participation in schools. Using wearable devices, early detection of abnormal signals in the biometric information of children with ASD, and conversion of problem behavior to prediction, mitigation, or alternative behaviors in advance will help improve children’s sociality.

The following are the objectives of this study: (1) developing a metaverse-based youth social skills training program using digital technology to improve the social interaction skills of children with ASD; (2) validating the effectiveness of the program developed as a metaverse for “Being a Good Sportsman,” which is part of the PEERS program, as well as validate the improvement of social interaction skills required by children with ASD; and (3) analyzing biometric information collected through wearable devices to confirm emotional changes in children with ASD during stressful situations.

## Methods

### Study Design

#### Summary

This study is a parallel randomized controlled study (trial registration: KCT0006859) involving children aged 7-12 years diagnosed with ASD who volunteered to participate in the study with the consent of a legal guardian. The study will include an intervention group and a control group at a 1:1 ratio. Participants will be publicly recruited through the internet to enroll in a clinical trial through the Korean-Wechsler Intelligence Scale for Children-IV (K-WISC-IV) test administered by a professional clinical psychologist. Following neuropsychological evaluation, the children eligible for this study will be assigned randomly to the intervention or the control group for 4 weeks. Randomization will be performed using the PROC PLAN method in the SAS software program (version 9.4, SAS Institute). In this study, we plan to develop and apply a social skills training program for children with high-function ASD that can be implemented within the metaverse platform based on the PEERS program using digital technology. The PEERS is a social skills training program developed for children with ASD who face difficulties in making or retaining friends. The PEERS program has 14 sessions. The 1st session gives the introduction and trading information; the 2nd session is about conversational skills; the 3rd session is on electronic communication; the 4th session is about choosing appropriate friends; the 5th session is regarding the appropriate use of humor; the 6th session is about peer entry strategies; the 7th session is regarding peer exit strategies; the 8th session is about get togethers, the 9th is on good sportsmanship; the 10th is about handling teasing; the 11th is on handling bullying and bad reputations; the 12th is regarding arguments and disagreements; the 13th is about handling rumors and gossip; and the 14th one includes a graduation party and ceremony [12]. Under the guidance of a clinical psychologist (with a PhD in education) and a clinical specialist in psychiatry, “Becoming a Good Sportsman,” which is part of the PEERS program, was selected and conducted as a proof-of-concept study. Socializing with peers is important for children and adolescents with ASD to build close friendships and engage in positive peer interactions. To reduce the burden of discussing a wide range of topics, it is better to proceed with activities that go well together. “Becoming a Good Sportsman” was selected because experts considered it suitable for a test study because it is composed of activity-based socializing to become a good sportsman, which corresponds to the ninth session selected in our study [26]. To maintain the consistency of the program, a professional clinical psychologist trained during the study period will conduct it. To verify the effectiveness of the program, the results of neuropsychological evaluation, including social development indicators and biometric information (heart rate, heart rate variability, saturation, respiratory rate, and stress index), will be collected through wearable devices. Children enrolled in the study will be randomized into 2 groups with the same baseline characteristics. To verify the effectiveness of the program, for each group, first the differences between the neuropsychological evaluation results, including the social development index determined before the experiment and the test results after the experiment, will be compared and analyzed using a statistical method. Second, the differences in the neurophysiological scores between the groups will be compared and analyzed. Based on the results of these 2 processes, the effectiveness of the metaverse-based social skills training program using digital technology will be verified.

#### Neuropsychological Test

To confirm that the children who wished to participate in the study meet the research criteria, the K-WISC-IV test, an intelligence test that comprehensively evaluates the overall intellectual ability of children, will be performed before the start of the study [27]. To compare and evaluate the effectiveness of the metaverse-based social skills training program developed in this study, a series of neuropsychological tests will be performed before and after the study to evaluate sociability. Neuropsychological tests to be used in the study include the Social Responsiveness Scale (SRS), evaluated by parents, as a tool to verify the effectiveness of children's social interactions. Furthermore, the Korean version of the Child Behavior Checklist (K-CBCL) and the Korean version of the Vineland Adaptive Behavior Scale-2 (K-VABS-2) test are used as evaluation tools to verify the children's overall problematic behavior, adaptability, and sociability-related effects as determined through a parental survey. The Children’s Depression Inventory (CDI) and revised Children’s Manifest Anxiety Scale (RCMAS) are used as assessment tools to compare mental health levels, such as stress relief, depression, and anxiety before and after intervention.

#### Biometric Information

During the 4 weeks of this study, all children participating in the study will use a wearable device shaped like a smartwatch (Fitbit) to minimize discomfort. In addition to collecting the children's biometric information using the smartwatch, a webcam will be used to record the children's behavior and facial expressions in real time during the program. The biometric information of the children with ASD will be compared and analyzed using the internet-based (metaverse) program with the biometric information of children with ASD and real-time biometric information recorded daily. Based on the study results, the effectiveness of the metaverse-based social skills training program using digital technology will be verified, and the children's anxiety and stress levels will be measured. The improvement of social interaction abilities has an important effect on children's adaptation to school life; therefore, its effect on improving mental health while alleviating stress and anxiety will also be verified.

#### Intervention Group Receiving the Metaverse-Based Social Skills Training Program

The intervention group will receive the metaverse-based social skills training program developed by the researchers for 60 minutes per session once a week for 4 weeks. The program consists of direct instruction (rules for being a good sportsman) and practical training (playing sport games). Table 1 summarizes the metaverse-based social skills training program content. When the program is applied to metaverse, a wearable device will collect the child's biometric information in real time. In addition to internet-based and offline recording modes, the children's actions will be recorded using a webcam during the program.

**Table 1:** Contents of the metaverse-based social skills training program.

Week	Session	Goal	Content
1	Introduction and preneuropsychological test (offline)	Orient the participants to the program	Explaining the process involved in the program and the basic rules and the use of the wearable device Preneuropsychological evaluation
2	Being a Good Sportsman 1(internet): awareness of the need for rules and results	Learn and develop the right behavior for replacing inappropriate behavior; avoid uncontrolled negative behavior to achieve positive behavior	Subject and modulator introducing themselves to each other Self-introduction by team Forming rules Playing sports games in metaverse Giving session feedback and homework
3	Being a Good Sportsman 2 (internet): understanding the situation and participating in team activities	Learn social understanding by noticing situations and conflicts that occur during team activities	Greeting therapists and participants Checking the homework from the last session Reminding rules Playing sports games in metaverse Giving session feedback and homework
4	Being a Good Sportsman 3 (internet): responding appropriately to negative behavior experiences and negative emotions	Learn to accept the result of rule violations, failures, etc experienced in team activities and understand appropriate emotional response	Greeting therapists and participants Checking the homework from the last session Educating and practicing negative emotion acceptance and coping skills Playing sports games in metaverse Giving session feedback and homework
5	Being a Good Sportsman 4 (internet): knowing and accepting individual differences	To be able to recognize the characteristics of other team members and accept similarities and differences with friends while working as a team	Greeting therapists and participants Checking the homework from the last session Educating and practicing “knowing and accepting” skills. Playing sports games in metaverse Giving session feedback
6	Neuropsychological test (offline)	Conducting postneuropsychological evaluation	Postneuropsychologital evaluation: K-SCQ^a^ SRS 2^b^ K-CBCL^c^ K-VABS-2^d^ SCL-R^e^ CDI^f^ RCMAS^g^

^a^K-SCQ: Korean Version of the Social Communication Questionnaire.

^b^SRS-2: Social Responsiveness Scale-2

^c^K-CBCL: Korean version of the Child Behavior Checklist.

^d^K-VABS-2: Korean version of the Vineland Adaptive Behavior Scale-2.

^e^SCL-R: Symptom Checklist-Revision.

^f^CDI: Children’s Depression Inventory.

^g^RCMAS: revised Children’s Manifest Anxiety Scale

### Control Group

The control group will not intervene. Only neuropsychological tests will be conducted between the first and last week.

### Measures

#### K-WISC-IV Test

The K-WISC-IV is used to establish a baseline for the participating children. This test evaluates the overall cognitive function of children using 15 subtests, such as common sense, missing places, and vocabulary. The K-WISC-IV is an individual test tool used to evaluate the cognitive abilities of children aged 6-16 years [27]. The evaluation items include the verbal comprehension index (VCI), visual spatial index (VSI), fluid reasoning index (FRI), working memory index (WMI), and processing speed index (PSI) that are combined to evaluate intellectual ability. The VCI is a measure of crystallized intelligence. It measures a child’s ability to access and apply acquired word knowledge. The VSI measures a child’s ability to evaluate visual details and understand visual spatial relationships to construct geometric designs from a model. The FRI measures a child’s ability to detect the underlying conceptual relationship among visual objects and use reasoning to identify and apply rules. The WMI measures a child’s ability to register, maintain, and manipulate visual and auditory information in conscious awareness. The PSI measures a child’s speed and accuracy of visual identification, decision-making, and decision implementation. It provides composite scores representing the overall intelligence quotient (IQ) as well as subtests and composite scores representing intellectual functioning in specific cognitive domains [28].

#### Korean Version of the Social Communication Questionnaire (K-SCQ)

The SCQ is a tool that clinicians use when screening individuals for ASD. The parent or primary caregiver of the target child can easily answer “yes/no” and screen a wide range of symptoms related to autism in a short time. It was designed as a questionnaire version of Autism Diagnostic Interview-Revised (ADI-R) [29]. It is a screening tool consisting of 40 items asking parents or caregivers about their children's ASD-related symptoms (communication, reciprocal interactions, and restrictive and repetitive behaviors and interests). There are 2 forms, namely the Lifetime AutoScore Form and the Current AutoScore Form. The Lifetime Form provides an answer based on the individual’s overall development, and the Current Form provides answers based on the individual's behavior in the last 3 months. The K-SCQ was translated into English and approved by the authors [30].

#### Social Responsiveness Scale-2 (SRS-2)

The SRS-2 is an evaluation tool used to verify the effectiveness of social interactions. This test is a questionnaire that asks parents or teachers to evaluate the characteristics of social interactions that children have displayed in the past 6 months and consists of 65 items. Each question is rated from “not at all” (0 points) to “almost always” (3 points) and can be scored on a scale of 0-195 points. The evaluation content consists of social insight, social information processing, mutual social communication ability, social anxiety/avoidance, and autistic immersion and traits. Higher scores indicate lower social functioning [31].

#### K-CBCL Evaluation

The K-CBCL is an evaluation tool used to verify the effects related to overall problem behavior, adaptation, and social performance. This test is a standardized checklist in which parents describe their children’s behavioral and emotional problems. It consists of two parts: the Social Ability Scale and the Syndrome and Total Problems Scale [32]. The K-CBCL consists of a 132-item questionnaire, and responses are provided on a 3-point Likert scale ranging from 0 to 2. It consists of a social competence scale, school performance scale, syndrome scale, and a total problem scale [32].

#### K-VABS-2 Analysis

The K-VABS-2 is an evaluation tool to verify the effects related to overall problem behavior, adaptation, and social performance. This test is the Korean version of the second edition of the VABS. It measures adaptive behavior, including 4 domains (communication, daily living skills, socialization, and motor function) and 11 subdomains (receptive language, expressive language, writing, individual, family, community, interpersonal relationship, play, ability to cope, and large and small muscle areas). This was evaluated by dividing the adaptation levels into 5 and maladaptation levels into 3 [33].

#### Symptom Checklist-Revision (SCL-R)

The SCL-R is a symptom checklist developed by Derogatis et al. It consists of 9 symptom dimensions (somatization, obsessive-compulsive disorder, interpersonal sensitivity, depression, anxiety, hostility, phobic anxiety, paranoid ideation, psychoticism, and additional items) with a total of 90 items [34].

#### CDI Evaluation

The CDI is an evaluation tool to verify the effect of improving mental health, such as relieving stress and reducing depression in children. This test measures the degree of depression in children. It is a modified version of the Adult Beck Depression Inventory (BDI) for children aged 8-13 years [35]. It consists of 27 items. The evaluation is provided in a self-report format, where the mood state of the individual is indicated in 1 of the 3 sentences describing each question [36].

#### RCMAS Evaluation

The RCMAS is an evaluation tool for testing mental health–enhancing effects, such as stress relief and anxiety. This tool is a children's version of Taylor's Manifest Anxiety Scale for Adults, and it is the most widely used self-report scale for the assessment of anxiety disorders in children and adolescents aged 0-19 years [37]. It is designed to evaluate various symptoms related to anxiety, and all 37 questions are asked to be answered with “yes” or “no” about how you think and feel about yourself.

### Recruitment

#### Ethics Approval and Consent to Participate

This study was approved by the Korea National Institute for Bioethics Policy (KONIBP), a Public Institutional Review Board (P01-202202-01-017). Before participating in the study, consent will be obtained from parents and participants (children).

### Participants

#### Setting

The intervention will be administered remotely from a treatment center for developing the social abilities of children with ASD in Korea or by self-referral through community web pages on the internet for parents with ASD.

#### Screening and Inclusion Criteria

We will recruit 24 children between March 2022 through treatment centers or internet-based social networking services. Inclusion criteria for children are the following: (1) age between 7 and 12 years, (2) diagnosed with ASD, (3) sufficient cognitive abilities to understand the rules with IQ≥90 according to a standardized intelligence test, (4) children and parents fluent in Korean, and (5) no defects in motor function. Written informed consent will be obtained from all the children and parents who will participate in this study.

#### Exclusion Criteria

Children will be excluded from the study if they do not speak, hear, or have impaired vision; if they have been diagnosed with a history of congenital or acquired brain damage, such as cerebral palsy; or if they have difficulty in cooperating with program participants because of serious developmental delays or difficulties in controlling behavior.

#### Sample Size

The sample size calculation provided by the clinical trial pilot test was employed because no studies were undertaken in advance to determine the effectiveness of the peer program by conducting exploratory clinical trials [38]. The recommended sample size for such pilot studies is 12 persons per group.

#### Randomization and Masking

Participants will be assigned (1:1) to either the experimental or control group using permuted blocks (block size 4) and stratified by age (children aged 7-9 years and 10-12 years). An independent statistician will build the randomization list with consecutive subject numbers using the PROC PLAN method in SAS version 9.4.

Researchers providing the interventions are not blind; however, those who perform the neuropsychological assessments of the participants are blinded. Because all participants will receive the same outcome measures, assessors will not be able to identify which group a participant belongs to, based on the results. Wherever possible, if an assessor becomes unblinded, further evaluations for that participant will be completed by a different assessor (blind to the arm allocation).

### Data Management

The data acquired in this study will not be viewed or leaked by anyone other than the researcher responsible for the participants’ safety, and participants’ data will be entered into the system when entering computerized data with security functions and kept confidential. Except for the information supplied by research institutes, data entered into the computerized data input system are inaccessible and are not accessed or leaked to anyone other than researchers licensed as accountable researchers. Furthermore, all research-related records will be kept for 3 years from the end of the study, per Article 15 of the Bioethics Act Enforcement Rules, and data relating to personal information among documents passed by the storage agency will be destroyed per Article 16 of the Personal Information Protection Act Enforcement Decree.

### Statistical Analysis

All baseline variables will be summarized in a randomized group. Continuous data will be reported as means (SD) unless skewed and will be reported as medians (IQR). Before testing the effectiveness of the program, an independent sample *t* test and a Mann-Whitney U test will be performed to determine whether there are differences in demographic variables between the groups. To compare the effects of all outcomes, the analysis will be conducted using the paired *t* test and Wilcoxon signed rank test. To evaluate treatment effects in each group, ANOVA will be repeatedly performed for determining differences in variables between the baseline and posttest for the treatment and control groups, with a condition analysis using (treatment vs control) × time (baseline vs posttest). ANOVA will be performed to compare the effects of the program between the groups and to determine whether there is a significant difference between each group by judging the rejection range based on a significance level of 0.05. We plan to perform statistical analysis using Python (version 3.8.5) and R (version 4.0.4).

## Results

We intend to recruit and enroll participants from March 2022. After registering the participants, the study will be conducted from March 2022 to May 2022, jointly by Yonsei University and Dobrain Co, Ltd. The recruited children will participate in internet-based programs. The results are scheduled to be published in July 2022.

## Discussion

### Significance of the Study

We expect that conducting the peer program on metaverse will improve the social skills of children with ASD. Studies have demonstrated that PEERS programs are significantly effective in improving the overall social skills, frequency of social engagement, and social skills knowledge, while reducing ASD symptoms. In addition, previous studies have shown the effectiveness of an additional 16-week follow-up observation, which can be interpreted as the program proving effective not only in terms of the effectiveness of the treatment but also in terms of persistence [10,39,40]. Our study is an RCT implementing “Being a Good Sportsman,” which is a part of the PEERS program in the VR world called metaverse. Numerous technical trainings to improve social skills have been conducted offline for practical purposes. We believe that using this offline technology in a metaverse setting will be just as helpful in enhancing sociality as using them offline, and that ASD children who struggled to adjust to their environment during offline education will be able to do so successfully in the metaverse environment. Metaverse can increase the scalability and freedom of the program in that it can provide the characteristics of surrounding people who can help ASD youth. In addition, if the program is conducted using metaverse rather than simply delivering education on the internet, it is possible to provide not only theoretical content but also practice using actual peer groups, thus enabling interactive learning. The internet-based “Becoming a Good Sportsman” program and this study are metaverse-based education programs, which include only some of the PEERS programs. This research can be expanded by developing and applying educational programs based on all PEERS programs.

When conducting the “Being a Good Sportsman” program on metaverse, a wearable device will be used to measure biometric information. Based on the biometric results, it is possible not only to verify the validity of internet-based programs but also to collect the children’s biometric information in specific situations (eg, anxiety, anger) and environments (eg, first visit place, home). By collecting data, we can detect children's behavior early and predict what they will do in the future. The collected biometric data can be used to address and alleviate children's anxiety in advance, thus significantly reducing the problematic behavior of children with ASD.

### Limitations

Computer equipment and internet connections can cause complications in a study using an internet-based metaverse game platform. Furthermore, because this is a pilot study, we are unable to generalize the effect of the metaverse-based social skills training program because of the small number of participants. We intend to evaluate the behaviors of the experimental and control groups shortly after intervention, because of which direct comparative tests of the metaverse-based social skills training program's long-term effectiveness will be impossible.

## Abbreviations

ASD: autism spectrum disorder
BDI: Beck Depression Inventory
CDI: Children’s Depression Inventory
FRI: fluid reasoning index
IQ: intelligence quotient
K-CBCL: Korean version of the Child Behavior Checklist
K-SCQ: Korean Version of the Social Communication Questionnaire
K-VABS-2: Korean version of the Vineland Adaptive Behavior Scale-2
K-WISC-IV: Korean-Wechsler Intelligence Scale for Children-IV
PEERS: Program for the Education and Enrichment of Relational Skills
RCMAS: revised Children’s Manifest Anxiety Scale
RCT: randomized controlled trial
SCL-R: Symptom Checklist-Revision
SCQ: Social Communication Questionnaire
SRS: Social Responsiveness Scale
SRS-2: Social Responsiveness Scale-2
VCI: verbal comprehension index
VSI: visual spatial index
VR: virtual reality
WMI: working memory index

## References

[1] Svenaeus F (2013). Diagnosing mental disorders and saving the normal. Med Health Care and Philos.

[2] Yoo H-J, Bahn G, Cho I-H, Kim E-K, Kim J-H, Min J-W, Lee W-H, Seo J-S, Jun S-S, Bong G, Cho S, Shin M-S, Kim B-N, Kim J-W, Park S, Laugeson EA (2014). A randomized controlled trial of the Korean version of the PEERS(®) parent-assisted social skills training program for teens with ASD. Autism Res.

[3] Gralinski JH, Kopp CB (1993). Everyday rules for behavior: mothers' requests to young children. Developmental Psychology.

[4] Ung D, Wood JJ, Ehrenreich-May J, Arnold EB, Fuji C, Renno P, Murphy TK, Lewin AB, Mutch PJ, Storch EA (2013). Clinical characteristics of high-functioning youth with autism spectrum disorder and anxiety. Neuropsychiatry (London).

[5] Baxter A (1997). The power of friendship. J Dev Disabl.

[6] Hong M, Lee SM, Park S, Yoon S, Kim Y-E, Oh I-H (2019). Prevalence and economic burden of autism spectrum disorder in South Korea using National Health Insurance data from 2008 to 2015. J Autism Dev Disord.

[7] Hyman SL, Levy SE, Myers SM (2019). Identification, evaluation, and management of children with autism spectrum disorder. Pediatrics.

[8] Whitehouse AJ (2013). Complementary and alternative medicine for autism spectrum disorders: rationale, safety and efficacy. J Paediatr Child Health.

[9] McPheeters ML, Warren Z, Sathe N, Bruzek JL, Krishnaswami S, Jerome RN, Veenstra-Vanderweele J (2011). A systematic review of medical treatments for children with autism spectrum disorders. Pediatrics.

[10] Laugeson EA, Gantman A, Kapp SK, Orenski K, Ellingsen R (2015). A randomized controlled trial to improve social skills in young adults with autism spectrum disorder: The UCLA PEERS® Program. J Autism Dev Disord.

[11] Laugeson EA, Frankel F, Mogil C, Dillon AR (2008). Parent-assisted social skills training to improve friendships in teens with autism spectrum disorders. J Autism Dev Disord.

[12] Laugeson EA, Frankel F, Gantman A, Dillon AR, Mogil C (2011). Evidence-based social skills training for adolescents with autism spectrum disorders: the UCLA PEERS Program. J Autism Dev Disord.

[13] Narzisi A (2020). Handle the autism spectrum condition during coronavirus (COVID-19) stay at home period: ten tips for helping parents and caregivers of young children. Brain Sci.

[14] Liu G, Wang S, Liao J, Ou P, Huang L, Xie N, He Y, Lin J, He H, Hu R (2021). The efficacy of WeChat-based parenting training on the psychological well-being of mothers with children with autism during the COVID-19 pandemic: quasi-experimental study. JMIR Ment Health.

[15] World Health Organization (2010). Telemedicine: opportunities and developments in member states: report on the second global survey on eHealth. WHO Global Observatory for eHealth.

[16] Heitzman-Powell LS, Buzhardt J, Rusinko LC, Miller TM (2013). Formative evaluation of an ABA outreach training program for parents of children with autism in remote areas. Focus Autism Other Dev Disabl.

[17] Bearss K, Burrell TL, Challa SA, Postorino V, Gillespie SE, Crooks C, Scahill L (2017). Feasibility of parent training via telehealth for children with autism spectrum disorder and disruptive behavior: a demonstration pilot. J Autism Dev Disord.

[18] Vismara LA, McCormick CEB, Wagner AL, Monlux K, Nadhan A, Young GS (2016). Telehealth parent training in the Early Start Denver Model: results from a randomized controlled study. Focus Autism Other Dev Disabl.

[19] Ke F, Im T (2013). Virtual-reality-based social interaction training for children with high-functioning autism. J Educ Res.

[20] Ip HHS, Wong SWL, Chan DFY, Byrne J, Li C, Yuan VSN, Lau KSY, Wong JYW (2018). Enhance emotional and social adaptation skills for children with autism spectrum disorder: a virtual reality enabled approach. Comput Educ.

[21] Kandalaft MR, Didehbani N, Krawczyk DC, Allen TT, Chapman SB (2012). Virtual reality social cognition training for young adults with high-functioning autism. J Autism Dev Disord.

[22] Ringland KE, Wolf CT, Dombrowski L, Hayes GR (2015). Making "safe": Community-centered practices in a virtual world dedicated to children with autism. CSCW '15: Proceedings of the 18th ACM Conference on Computer Supported Cooperative Work & Social Computing.

[23] Spiel K, Frauenberger C, Keyes O, Fitzpatrick G (2019). Agency of autistic children in technology research—a critical literature review. ACM Trans Comput-Hum Interact.

[24] Du Y, Grace TD, Jagannath K, Salen-Tekinbas K (2021). Connected play in virtual worlds: communication and control mechanisms in virtual worlds for children and adolescents. Multimodal Technol Interact.

[25] White SW, Mazefsky CA, Dichter GS, Chiu PH, Richey JA, Ollendick TH (2014). Social‐cognitive, physiological, and neural mechanisms underlying emotion regulation impairments: understanding anxiety in autism spectrum disorder. Int. J Dev Neurosci.

[26] Haddad J (2013). Social skills for teenagers with developmental and autism spectrum disorders:the PEERS treatment manual. J Child Adolesc Ment Health.

[27] Mayes SD, Calhoun SL (2007). WISC-IV and WIAT-II profiles in children with high-functioning autism. J Autism Dev Disord.

[28] Park H, Lee KO, Lee S-H, Park M (2016). A study on standardization of K-WPPSI-IV: analyses of reliability and validity. Korean J Early Child Educ.

[29] Lord C, Rutter M (2003). Social Communication Questionnaire (SCQ).

[30] Lord C, Rutter M, HeeJeong Y (2008). Korean version of Social Communication Questionnaire (SCQ).

[31] Constantino JN, Gruber CP (2012). Social Responsiveness Scale, Second Edition (SRS-2).

[32] Lee H, Oh KJ, Hong K-E, Ha EH (1991). Clinical validity study of korean CBCL through item analysis. J Korean Acad Child Adolesc Psychiatry.

[33] Hwang ST, Kim JH, Hong SH, Bae S, Cho S (2015). Standardization Study of the Korean Vineland Adaptive Behavior Scales-Ⅱ(K-Vineland-II). Korean J Clin Psychol.

[34] Derogatis LR (1977). SCL-90R: Administration, scoring and procedures manual for the revised version. Manual II for the R (evised) Version and Other Instruments of the Psychopathology Rating Scale Series.

[35] Saylor CF, Finch Jr AJ, Spirito A, Bennett B (1984). The Children's Depression Inventory: a systematic evaluation of psychometric properties. J Consult Clin Psychol.

[36] Cho SC, Lee YS (1990). Development of the Korean form of the Kovacs' Childeren's Depression Inventory. J Korean Neuropsychiatr Assoc.

[37] Reynolds CR, Paget KD (2019). National normative and reliability data for the Revised Children's Manifest Anxiety Scale. Sch Psychol Rev.

[38] Julious SA (2005). Sample size of 12 per group rule of thumb for a pilot study. Pharmaceut Statist.

[39] Gantman A, Kapp SK, Orenski K, Laugeson EA (2012). Social skills training for young adults with high-functioning autism spectrum disorders: a randomized controlled pilot study. J Autism Dev Disord.

[40] Mandelberg J, Laugeson EA, Cunningham TD, Ellingsen R, Bates S, Frankel F (2013). Long-term treatment outcomes for parent-assisted social skills training for adolescents with autism spectrum disorders: The UCLA PEERS Program. J Ment Health Res Intellect Disabil.

