# Correction: Development and Application of a Metaverse-Based Social Skills Training Program for Children With Autism Spectrum Disorder to Improve Social Interaction: Protocol for a Randomized Controlled Trial

**DOI:** 10.2196/43864

**Published:** 2022-11-14

**Authors:** JooHyun Lee, Tae Seon Lee, SeungWoo Lee, JiHye Jang, SuYoung Yoo, YeJin Choi, Yu Rang Park

**Affiliations:** 1 Department of Biomedical Systems Informatics Yonsei University College of Medicine Seoul Republic of Korea; 2 Department of Neurosurgery, Severance Hospital Yonsei University College of Medicine Seoul Republic of Korea; 3 Graduate School of Information and Communication Technology Ajou University Suwon Republic of Korea; 4 DoBrain Co, Ltd Seoul Republic of Korea

In “Development and Application of a Metaverse-Based Social Skills Training Program for Children with Autism Spectrum Disorder to Improve Social Interaction: Protocol for a Randomized Controlled Trial” (JMIR Res Protoc 2022;11(6):e35960) the authors made one addition.

In the originally published article, the Acknowledgments section appeared as follows:

This research was supported by a grant from the Korea Health Technology R&D Project through the Korea Health Industry Development Institute (KHIDI), funded by the Ministry of Health & Welfare, Republic of Korea (grant HI19C1015) and Institute of Information & communications Technology Planning & Evaluation (IITP) grant funded by the Korea government (MSIT).

The Acknowledgments section has been corrected to:

This work was supported by Institute of Information & communications Technology Planning & Evaluation (IITP) grant funded by the Korea government(MSIT) (No. 2022-0-00234, Development of digital therapeutics (DTx) on social interaction skills of patients with Autism Spectrum Disorder)

The correction will appear in the online version of the paper on the JMIR Publications website on November 14, 2022, together with the publication of this correction notice. Because this was made after submission to PubMed, PubMed Central, and other full-text repositories, the corrected article has also been resubmitted to those repositories.

